# Cyanobacteria as Cell Factories to Produce Plant Secondary Metabolites

**DOI:** 10.3389/fbioe.2015.00057

**Published:** 2015-04-28

**Authors:** Yong Xue, Qingfang He

**Affiliations:** ^1^Department of Applied Science, University of Arkansas at Little Rock, Little Rock, AR, USA

**Keywords:** plant secondary metabolites, phenylpropanoid, cyanobacteria, P450 proteins, photosynthetic growth

## Abstract

Cyanobacteria represent a promising platform for the production of plant secondary metabolites. Their capacity to express plant P450 proteins, which have essential functions in the biosynthesis of many plant secondary metabolites, makes cyanobacteria ideal for this purpose, and their photosynthetic capability allows cyanobacteria to grow with simple nutrient inputs. This review summarizes the advantages of using cyanobacteria to transgenically produce plant secondary metabolites. Some techniques to improve heterologous gene expression in cyanobacteria are discussed.

## Benefits of Plant Secondary Metabolites to Human Health

Secondary metabolites produced by plants confer protection against stresses such as infections, wounding, UV irradiation, and ozone (Douglas, 1996), and allow plants to adapt to continuously changing environmental conditions (Korkina, 2007). Secondary metabolites are largely derived from primary metabolites, such as amino acids and carbohydrates, which are modified by methylation, hydroxylation, or glycosylation (Crozier et al., 2006).

Increasing evidence suggests that plant secondary metabolites, especially the largest group, phenylpropanoids, and their derivatives, are powerful antioxidants that directly scavenge reactive oxygen and nitrogen species (ROS/RNS) (Perron and Brumaghim, 2009). A balance between oxidant and antioxidant systems is critical for maintaining cellular functions. The excessive production of ROS/RNS inside cells results in oxidative stress, loss of cell function, and apoptosis or necrosis. Detoxification of ROS/RNS by enzymatic and non-enzymatic antioxidants minimizes cell damage (Ratnam et al., 2006; Reuter et al., 2010). The antioxidant activity of five phenylpropanoids (i.e., verbascoside, forsythoside, arenareoside, ballotetraside, and caffeoyl malic acid) extracted from the perennial herb *Ballota nigra* were investigated against superoxide, hydrogen peroxide, hypochlorite, and hydroxyl radicals generated in cell-free systems (Fraga et al., 2010). The ability of these phenylpropanoids to scavenge free radicals was comparable to that of *N*-acetyl cysteine, an established antioxidant drug (Nordberg and Arner, 2001).

Plant secondary metabolites have become the focus of intensive research, due to their beneficial effects on human health as anticancer, antioxidant, anti-virus, and anti-inflammatory agents. However, these compounds are mainly isolated from plant extracts or from cultivated plant cells at relatively high cost and low yield. It is expensive and technically challenging to chemically synthesize these molecules. Therefore, there is a strong need to develop novel, efficient, and economical methods to produce beneficial plant secondary metabolites.

## Cyanobacteria are Suitable for Producing Plant Secondary Metabolites

Several inherent properties of cyanobacteria make them attractive candidates for the biosynthesis of plant secondary metabolites, such as their photosynthetic activity, their amenability to genetic engineering, and their ability to live in tough environments.

Large-scale cyanobacterial cultivation is frequently performed in phototrophic conditions because this approach is cheaper, results in less contamination, and consumes CO_2_ (Chen et al., 2011). Commercially used open ponds or closed photobioreactors have been developed for large-scale biomass production (Olaizola, 2000; López et al., 2006; Eriksen, 2008; Ugwu et al., 2008; Rodolfi et al., 2009; Singh and Gu, 2010). The nutrient inputs for cyanobacteria are simple: sunlight, CO_2_, H_2_O, N, P, and a few mineral nutrients, without carbohydrate feedstocks (Yu et al., 2013).

A wide variety of enzymes and pathways are involved in plant secondary metabolite production. Of these enzymes, cytochrome P450 monooxygenases participate in the pathways to produce compounds such as phenylpropanoids, alkaloids, terpenoids, cyanogenic glycosides, and glucosinolates (Mizutani and Ohta, 2010). They contribute various oxidative modifications of the carbon skeleton using NADPH or NADH as reducing equivalents (Sligar, 1999). P450 sequences have been found in the genome of most known cyanobacterial species (Ke et al., 2005); *Anabaena* sp. PCC 7120 has six P450 genes (Robert et al., 2010) and *Synechocystis* sp. PCC 6803 has one (*cyp120A1*/*slr0574*) (Ke et al., 2005). Because most eukaryotic P450 proteins are membrane-bound proteins, it is challenging to heterologously express these proteins in other prokaryotes, such as *E. coli*, which lack developed internal membrane systems. By contrast, cyanobacteria have an intracellular membrane system, i.e., the thylakoids, which function in electron transport. This makes cyanobacteria highly suitable hosts in which to express P450 enzymes (Melis, 1999).

## Using Cyanobacteria to Produce Plant Secondary Metabolites

**Figure 1:**
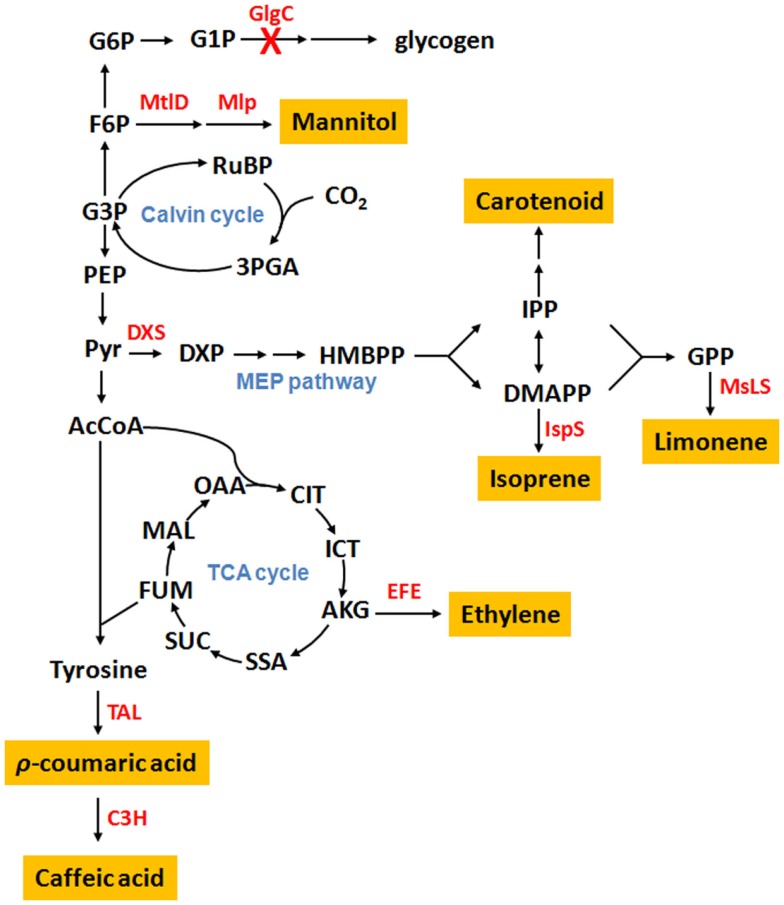
**Cyanobacterial metabolic pathways for the production of plant secondary metabolites**. Abbreviation: G6P, glucose-6-phosphate; G1P, glucose-1-phosphate; F6P, fructose-6-phosphate; G3P, glyceraldehyde-3-phosphate; RuBP, ribulose-1,5-diphosphate; 3PGA, 3-phosphoglycerate; PEP, phosphoenolpyruvate; Pyr, pyruvate; DXP, 1-deoxy-d-xylulose 5-phosphate; HMBPP, 4-hydroxy-3-methylbut-2-enyl diphosphate; IPP, isopentenyl pyrophosphate; DMAPP, dimethylallyl pyrophosphate; GPP, geranyl pyrophosphate; AcCoA, acetyl-CoA; OAA, oxoacetate; CIT, citrate; ICT, isocitrate; AKG, α-ketoglutarate; SSA, succinic semialdehyde; SUC, succinate; FUM, fumarate; MAL, malate; GlgC, ADP-glucose pyrophosphorylase; MtlD, mannitol-1-phosphate dehydrogenase; Mlp, mannitol-1-phosphatase; DXS, 1-deoxy-d-xylulose-5-phosphate synthase; IspS, isoprene synthase; MsLS, *Mentha spicata* limonene synthase; EFE, ethylene-forming enzyme; TAL, tyrosine ammonia-lyase; C3H, ρ-coumarate 3-hydroxylase; H.

Several cyanobacteria have been engineered as cell factories for the production of plant secondary metabolites (Table 1). Metabolic distributions of produced plant secondary metabolites are summarized in Figure 1 based on their biosynthetic pathways.

**Table 1:** **Plant secondary metabolites produced by genetically engineered cyanobacteria**.

Products	Yield	Host	Reference
Ethylene	~512 μg L^−1^ h^−1^ OD730^−1^^b^	*S. elongatus* PCC 7942	Takahama et al. (2003)
	~3.9 μg L^−1^ h^−1^ OD730^−1b^	*S. elongatus* PCC 7942	Jindou et al. (2014)
Isoprene	50 μg (g dry cell)^−1^ day^−1^	*Synechocystis* sp. PCC 6803	Lindberg et al. (2010)
	~125 μg (g dry cell)^−1^ day^−1^	*Synechocystis* sp. PCC 6803	Bentley et al. (2014)
Caffeic acid^a^	7.2 mg L^−1^	*Synechocystis* sp. PCC 6803	Xue et al. (2014b)
ρ-coumaric acid^a^	82.6 mg L^−1^	*Synechocystis* sp. PCC 6803	Xue et al. (2014a)
Mannitol	0.15 g L^−1^ day^−1^	*Synechococcus* sp. PCC 7002	Jacobsen and Frigaard (2014)
Limonene	56 μg L^−1^ day^−1^	*Synechocystis* sp. PCC 6803	Kiyota et al. (2014)
	50 μg L^−1^ h^−1^	*Synechococcus* sp. PCC 7002	Davies et al. (2014)
Carotenoid^a^	8.4 mg (g dry cell)^−1^	*Synechocystis* sp. PCC 6803	Kudoh et al. (2014)

*^a^Time over which the yield was achieved is not provided in the publication*.

*^b^The yield is calculated from ethylene gas production of 451 nL mL^−1^ h^−1^ OD370^−1^ reported by Takahama et al. (2003). The authors believe that the rate of 37 mg L^−1^ h^−1^ previously attributed to this study (Lan and Liao, 2011; Shen and Liao, 2012; Wang et al., 2012) is incorrect*.

## Tricarboxylic Acid Cycle

To produce ethylene, an ethylene-forming enzyme gene (efe) from *Pseudomonas syringae* was inserted into the *Synechococcus elongatus* PCC 7942 chromosome at the psbAI locus, and the recombinant strain produced ~512 μg ethylene L^−1^ h^−1^ OD730^−1^ (Takahama et al., 2003). An artificial chimeric enzyme complex containing two ethylene-generating enzymes from *Solanum lycopersicum* (tomato) was introduced into *S. elongatus* PCC 7942, and the strain produced ethylene with a titer of ~3.9 μg ethylene L^−1^ h^−1^ OD730^−1^ (Jindou et al., 2014).

## 2-C-Methyl-d-Erythritol 4-Phosphate Pathway

Isoprene, which protects plants from abiotic stresses (Sharkey et al., 2008) and serves as a renewable biofuel, was produced in *Synechocystis* sp. PCC 6803 with a titer of 50 μg (g dry cell)^−1^ day^−1^ by expressing an isoprene synthase gene (*ispS*) from *Pueraria Montana* (Lindberg et al., 2010). By co-expressing seven genes of a heterologous mevalonic acid biosynthetic pathway from *Enterococcus faecalis* and *Streptococcus pneumoniae* in that *ispS* transformant, the yield of isoprene production increased 2.5-fold (Bentley et al., 2014). To produce limonene, a limonene synthase gene (*LMS*) from *Schizonepeta tenuifolia* was expressed in *Synechocystis* sp. PCC 6803, and the titer was 41 μg L^−1^ day^−1^. By overexpressing three genes (*dxs*, *crtE*, and *ipi*) in the 2-C-methyl-d-erythritol-4-phosphate (MEP) pathway to increase the supply of limonene substrate, geranyl pyrophosphate (GPP), limonene production was improved by 1.4-fold (Kiyota et al., 2014). In another study, 50 μg L^−1^ h^−1^ limonene was produced by a *Synechococcus* sp. PCC 7002 strain expressing codon optimized *Mentha spicata* limonene synthase gene (*mslS*) (Davies et al., 2014). Carotenoids are naturally produced terpenoid-type molecules by cyanobacteria through MEP pathway. Recently, Kai et al. overexpressed a key enzyme, 1-deoxy-d-xylulose-5-phosphate synthase (DXS), in *Synechocystis* sp. PCC 6803, and the carotenoid level in the strain was 1.5 times higher than that in the wild-type strain (Kudoh et al., 2014).

## Other Metabolites

Our group recently constructed a *Synechocystis* sp. PCC 6803 strain genetically engineered to produce caffeic acid, in which an *Arabidopsis* ρ-coumarate 3-hydroxylase (encoded by *ref8*) was expressed. With the addition of substrate, ρ-coumaric acid, to the medium, the titer reached 7.2 mg L^−1^ (Xue et al., 2014b). In another report, the direct precursor of caffeic acid, ρ-coumaric acid, was produced at a concentration of 82.6 mg L^−1^ by a *Synechocystis* sp. PCC 6803 mutant, which harbored a tyrosine ammonia-lyase (TAL) gene (*sam8*) from *Saccharothrix espanaensis* and lacked a hypothetical laccase gene (Xue et al., 2014a). Mannitol was produced in *Synechococcus* sp. PCC 7002 with a titer of 0.15 g L^−1^ day^−1^ by heterologously expressing mannitol-1-phosphate dehydrogenase (*mtlD*) from *E. coli* and mannitol-1-phosphatase (*mlp*) from *Eimeria tenella*. By genetically inactivating glycogen synthesis, the yield increased 3.2-fold (Jacobsen and Frigaard, 2014).

## Some Considerations for the Genetic Manipulation of Cyanobacteria

Although many species of cyanobacteria have been successfully engineered to express heterologous genes and produce valuable compounds, as summarized above, this approach remains challenging due to epigenetic suppression, poor transcription, and protein post-translational modifications. Here, we highlight some factors that need to be considered when genetically engineering cyanobacteria to produce plant secondary metabolites.

## Inactivation of Glycogen Synthesis Pathway

Glycogen is one of the dominant carbon sinks for cyanobacteria. Up to 60% of dry cell weight is converted to glycogen when cyanobacteria are growing in nitrogen-limited media (Allen and Smith, 1969). Therefore, inactivation of glycogen synthesis pathway should allow a greater proportion of carbon partitioning to non-native products. In a recent study, the *glgC* gene (coding for glucose-1-phosphate adenylyltransferase) was knocked out from *S. elongatus* PCC 7942 strain producing isobutanol. The amount of total fixed carbon flux toward isobutanol production was increased by 2.5-fold after deletion of *glgC* under constant high light condition (150 μE s^−1^ m^−2^) (Li et al., 2014). Another example is a *Synechoccocuus* sp. PCC 7002 strain expressing a mannitol biosynthetic pathway, which produces mannitol equivalent to 10% of cell dry weight under 250 μE s^−1^ m^−2^. By blocking glycogen biosynthesis, the yield increased to 32% (Jacobsen and Frigaard, 2014).

## Laccase Knockout

Laccases are enzymes that oxidize many phenolic compounds and are present in a large variety of species. Oxidized substrates become free radicals, which are unstable and can be modified by other non-enzymatic reactions, such as hydration, polymerization, and disproportionation (Thurston, 1994). In a recent study, a putative laccase gene, *slr1573*, was found in *Synechocystis* sp. PCC 6803. After knocking out *slr1573* from its genome, the production of *p*-coumaric acid increased by more than 25-fold in the strain expressing TAL enzyme (Xue et al., 2014a). Although reports of laccase functions in cyanobacteria are limited, this gene might be a barrier for phenylpropanoids production using cyanobacteria.

## Codon Optimization

A codon usage bias exists for most protein-coding genes expressed in heterologous hosts. It is critical to optimize the codon usage in order to obtain high levels of overexpression of heterologous genes (Steen et al., 2010; Bond-Watts et al., 2011; Paddon et al., 2013). Several factors need to be considered when designing new gene sequences, including host codon usage frequency (Angov et al., 2011), AT/GC ratio (Gustafsson, 2009), mRNA secondary structure (Tang et al., 2011), repeat sequences (Li et al., 2011), and restriction sites for cloning (Raab et al., 2010). Our group recently constructed a transgenic *Synechocystis* sp. PCC 6803 that heterologously expressed *ref8* from *Arabidopsis thaliana*, which encodes a P450 enzyme ρ-coumarate 3-hydroxylase, and was capable of producing caffeic acid (Xue et al., 2014b).

## Transgene Stability

Several reports describe the instability of transgenes in genetically engineered cyanobacteria. During subculturing of an ethylene-producing *S. elongatus* PCC 7942 transformant, a duplicated sequence in the *efe* gene was found that resulted in a truncated and non-functional gene (Takahama et al., 2003). In another study, a *Synechocystis* sp. PCC 6803 strain was genetically modified to produce lactic acid by integrating a lactate dehydrogenase gene in the genome. Wild-type phenotypic colonies appeared during segregation, and further analysis identified a nonsense mutation in the transgene (Angermayr et al., 2012). Similarly, in an effort to produce isopropanol, four enzymes in the isopropanol biosynthetic pathway from *Clostridium acetobutylicum*, *Clostridium beijerinckii*, and *E. coli* were expressed in *S. elongatus* PCC 7942. The authors repeatedly found a missense mutation in one gene (*atoD*), which reduced the enzymatic activity (Kusakabe et al., 2013). The mechanism underlying the instability has not been determined. A common approach is to select transformants carrying single unrearranged transgenes and to keep these as backups.

## Markerless System

The standard method of genetically engineering cyanobacteria involves transformation of a plasmid carrying genes of interest and integration of these foreign genes into the cyanobacterial genome at specific sites through double-crossover homologous recombination (Vermaas, 1996). Antibiotic resistances are used as selectable markers for positive transformants. In some cases, when multiple gene integrations are required in one strain, the number of available antibiotic markers restricts the number of insertions, and thus markerless genomic mutations are desirable. Currently, the most widely used markerless technique for cyanobacteria was developed using a plasmid containing *sacB* (a levansucrase gene) and an antibiotic resistance cassette (Lagarde et al., 2000). In the first transformation, a target region in the genome is replaced by the *sacB*-antibiotic resistance cassette and antibiotic resistance is used for positive selection. The second transformation is performed by replacing the *sacB*-antibiotic resistance cassette with the gene of interest. Sucrose is added to the medium for negative selection. The levansucrase encoded by *sacB* converts sucrose to levans, a toxic polymer that kills the bacteria. Consequently, only markerless mutants can survive in the presence of sucrose. For example, a *Synechocystis* sp. PCC 6803 mutant was constructed for ethanol production by integrating pyruvate decarboxylase (*pdc*) and alcohol dehydrogenase II (*adh*) genes into the genome. An *aphX/sacB* selection cassette was used to generate a markerless transformant, which is able to produce ethanol at a titer of 5.2 mmol OD730 unit^−1^ L^−1^ day^−1^ (Dexter and Fu, 2009). Recently, an alternative strategy was developed using a one-step gene replacement approach (Viola et al., 2014). The plasmid designed for this strategy harbors an *nptI* (kanamycin resistance gene)-*sacB* selection cassette flanked by 5′and 3′ fragments of the gene of interest, which have overlapping segments. After transformation, the *nptI-sacB* cassette with the exogenous gene is integrated into the genome through double-crossover recombination, and complete segregations are selected based on kanamycin resistance. Then, a second single crossover event occurred between the overlapping fragments, leading to the excision of the *nptI-sacB* cassette. Mutants that had undergone the second recombination were screened on sucrose in the absence of kanamycin.

Another successful counter selection method based on acrylate toxicity was developed for *Synechococcus* sp. PCC 7002, in which *sacB* counter selection system did not work (Begemann et al., 2013). After one transformation step, a native *acsA* gene (encoding an acetyl-CoA ligase) is replaced by the gene of interest, and the loss of acetyl-CoA ligase function overcomes growth inhibition by acrylate. Thus, positive transformants can be screened on growth medium with addition of acrylate. After reinsertion of the *acsA* gene into a neutral site on genome, multiple gene integrations can be achieved.

## Limitations of Cyanobacteria for Producing Plant Secondary Metabolites

In addition to the potential advantages of using cyanobacteria to produce plant secondary metabolites mentioned above, this technology is still in its infancy and numerous challenges need to be addressed. For instance, production titers from engineered cyanobacteria are much lower than that from heterotrophic fermentation, and efficient, large volume bioreactors need to be designed. Another consideration is the protein post-translational modifications. Because some plant enzymes in the secondary metabolite biosynthesis pathways require post-translational modifications (e.g., glycosylation), they could be non-functional when expressed in cyanobacteria that are not equipped with these machineries.

## Conclusion

Cyanobacteria can be used as cell factories to convert solar energy into high value products, such as plant secondary metabolites, which are beneficial to human health. Their high photosynthetic efficiency and ease of genetic manipulation make cyanobacteria a better choice for this purpose than other organisms. Recently, researchers have put efforts into engineering cyanobacteria to produce plant secondary metabolites from sunlight and CO_2_. However, there are still challenges for engineering applications of cyanobacteria, such as improvement of product titers, bioprocess scale-up, and product recovery.

## Conflict of Interest Statement

The authors declare that the research was conducted in the absence of any commercial or financial relationships that could be construed as a potential conflict of interest.
